# Promoting engineering students’ social responsibility and willingness to act on socioscientific issues

**DOI:** 10.1186/s40594-023-00402-1

**Published:** 2023-02-09

**Authors:** Yohan Hwang, Yeonjoo Ko, Sungok Serena Shim, Seung-Yong Ok, Hyunju Lee

**Affiliations:** 1 College of General Education, Seoul Women’s University, 621 Hwarangro, Nowon-gu, Seoul, 01797 Republic of Korea; 2 Research Center for Hazard Literacy Education, Ewha Womans University, 52 Ewhayeodae-gil, Seodaemun-gu, Seoul, 03760 Republic of Korea; 3 Department of Educational Psychology, Ball State University, 2000 W. University Ave, Muncie, IN 47306 USA; 4 School of Social Safety System Engineering & Research Center for Safety and Health, Hankyong National University, 327 Jungang-ro, Anseong-si, Gyeonggi-do 17579 Republic of Korea; 5 Department of Science Education, Ewha Womans University, Rm. 419, College of Education Building A, 52 Ewhayeodae-gil, Seodaemun-gu, Seoul, 03760 Republic of Korea

**Keywords:** Social responsibility, Engineering students, Instructional model, Socioscientific issues

## Abstract

**Background:**

Despite increasing awareness of the importance of promoting the social responsibility of science, technology, engineering, and mathematics (STEM) professionals, few intervention programs have been developed to enhance the social responsibility of college students or adults in the STEM fields. In this paper, we introduced a new instructional program, called ENACT (engage, navigate, anticipate, conduct, and take action) and examined whether the program increased the social responsibility among safety engineering students (*N* = 46) recruited from a university located in a southern metropolitan area of South Korea.

**Results:**

In the ENACT program, the college students selected and explored socioscientific issues (SSIs) of their own interest then autonomously engaged in scientific and engineering group projects spanning a semester where they developed solutions to the SSIs and shared them with their communities. At the conclusion of the intervention in this study, they displayed an increased social responsibility regarding the consideration of societal needs and demands, civic engagement and services, and participation in policy decision-making. Social responsibility scores measured after the intervention (post-test) correlated with students' willingness to voluntarily participate in projects involving SSIs. In addition, the intervention effects were more pronounced for the students who initially had medium and low social responsibility scores.

**Conclusions:**

We have shown that social responsibility can be nurtured by systemic instructional approaches, and increased social responsibility can lead to greater commitment to resolving SSIs. Mastering engineering content knowledge and skills is the key element of engineering curricula. However, we are compelled to incorporate social responsibility into the STEM curriculum. We believe that the ENACT model contributes toward this end.

## Introduction

As Beck (1992) noted, we are living in a “risk society”, in which scientific and technological advances constantly create new forms of risks such as infectious diseases, exposure to dangerous chemicals, environmental pollution, sinkholes, and energy shortages. These risks involve socioscientific issues (SSIs), which refer to socially relevant, real-world scientific problems that often include an ethical component (Sadler et al., 2007). Many scholars and educators have insisted that scientists and engineers should pay more attention to and feel responsible for these emerging risks and issues (Eijkelhof, 1986; Hansen & Hammann, 2017; Harris et al., 2013). Scientists and engineers cannot predict or monitor all risks, as uncertainty and uncontrollability are inherent in all areas of science and technology (Beck, 1992). Uncertainty is particularly prominent in new technologies such as nanoscience or biotechnology. Thus, it is unfair to say that negative societal consequences are entirely due to the lack of responsibility of scientists and engineers.

Nonetheless, considering the occurrence of pervasive risk, STEM professionals' responsibilities cannot be completely waived. Thus, STEM professionals must be aware of and make an effort to reduce the potential risks of scientific discoveries and applications (Bielefeldt, 2018; Wyndham et al., 2015). Additionally, they have expert knowledge and skills in their respective areas that may not be as accessible to the general public. With such expertise and privileged vantage points, they are in a good position to predict the impact that scientific discoveries and engineering applications can have on humans, the environment, and society. As such, the collective body of STEM professionals can guide these efforts to minimize the risk and the negative consequences that can result from societal advances.

Although there has been an increasing awareness of the importance of promoting the social responsibility of STEM professionals, few intervention programs have been designed to enhance the social responsibility of college students or adults in STEM fields (Mejlgaard et al., 2019; Tassone et al., 2018; Zandvoort et al., 2013). This is problematic, as the social responsibility of STEM professionals does not seem to naturally emerge or develop over time without explicit efforts to raise it. For example, Bielefeldt and Canney (2016) examined the changes in engineering students’ social responsibility over time at five institutions and found that the majority of the students (57%) did not show significant changes and some even showed declines in social responsibility scores.

Many colleges of engineering in South Korea have tried to reorganize curricula for this purpose. Many engineering courses aim to address societal problems using problem- or project-based methods. In general, these courses tend to approach the problems from engineering perspectives, which mostly focus on how to solve the problems effectively and creatively using engineering skills and techniques (Jang et al., 2013). Although these efforts are laudable, these approaches do not seem sufficient to cultivating students’ social responsibility. Scholars recommend that students should be provided with enough opportunities to consider the societal and environmental impacts of science and engineering and to collaborate with diverse stakeholders to bring in non-engineering elements (Downey et al., 2006; Payne & Jesiek, 2018; Tassone et al., 2018; Zandvoort et al., 2013).

**Fig. 1:**
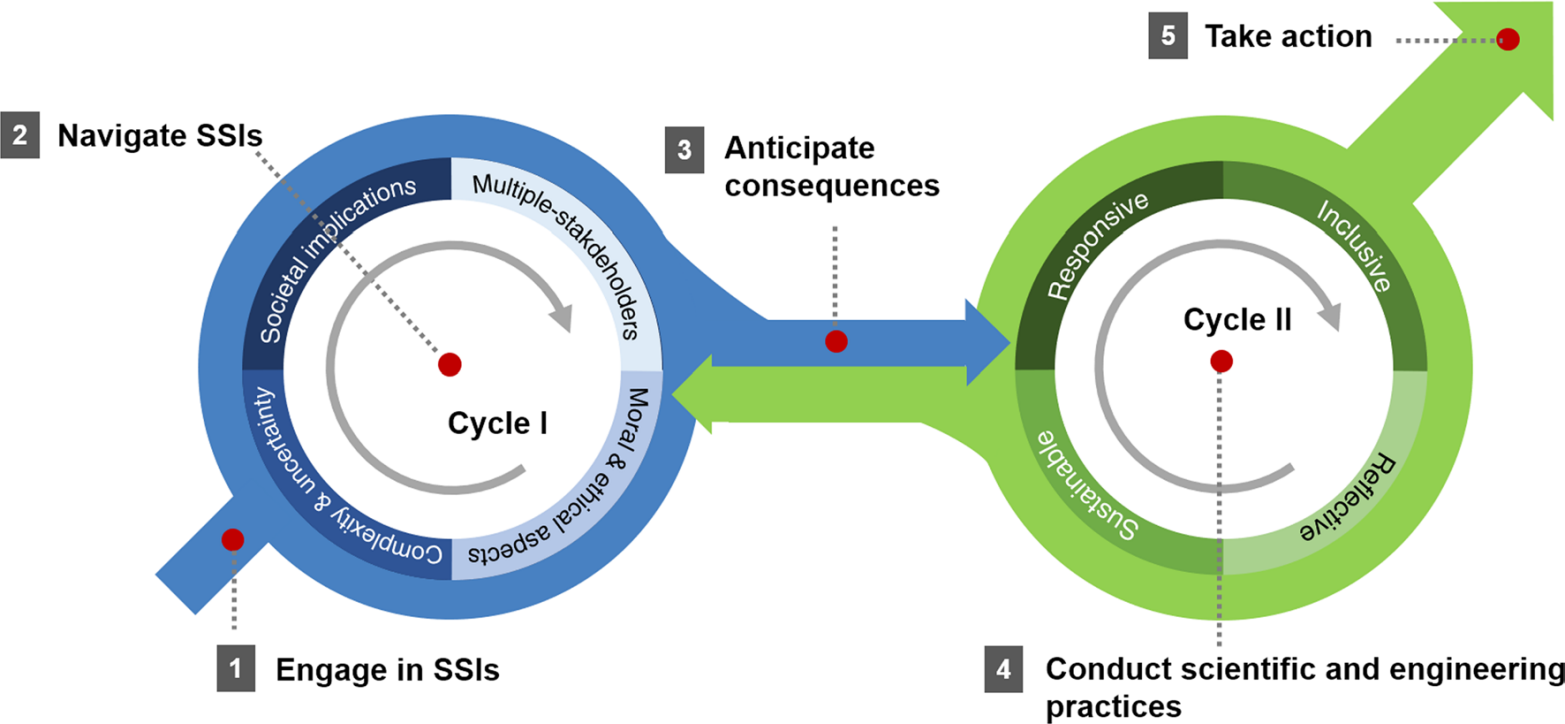
ENACT model

In the line of the efforts, we propose a new instructional approach, called the ENACT model, to enhance engineering students’ social responsibility and willingness to act to mitigate risks and other societal issues. “ENACT” is an acronym referring to the five steps of students’ investigation (*e*ngage in SSIs, *n*avigate SSIs, *a*nticipate consequences, *c*onduct scientific and engineering practices, and *t*ake action; see Fig. 1 for the full model; Lee et al., 2020). The ENACT model depicts the learning process in which college students select and explore SSIs based on their own interests, autonomously perform scientific and engineering group projects spanning a semester and share the solutions to the SSIs with their communities.

### The focus of the current investigation and research questions

The ENACT model shares some key elements with the prior interdisciplinary approaches. Students are encouraged to explore a broad range of knowledge (Zandvoort et al., 2013), to engage in the process of knowledge production by collaborating and communicating with diverse stakeholders (Payne & Jesiek, 2018; Tassone et al., 2018), and to participate in community services or volunteer activities (Bielefeldt & Canney, 2016). Thus, the ENACT intervention program, guided by the ENACT conceptual model, can yield many pedagogical benefits such as a broad understanding of content knowledge, the ability to work in a team, problem-solving skills, and awareness of societal issues. In the current study, our primary goal was to investigate whether the ENACT intervention can increase engineering students’ perception of social responsibility.

Toward this end goal, we first examined the changes in engineering students’ social responsibility, as measured by the eight factors included in the instrument to measure social responsibility, the Views of Social Responsibility of Scientists and Engineers (VSRoSE), developed by the authors (Ko et al., 2021). We also examined whether the social responsibility measured after participating in the semester-long intervention correlated with students' willingness to participate in workshops or projects involving SSIs. We tested these correlations to ensure that the increases in students’ social responsibility scores were related to the willingness to get involved in activities requiring social responsibility. Participating in these voluntary activities requires the students to invest time and effort and thus, the presence of such correlations will yield additional evidence that the intervention was effective.

Second, we identified the groups of students with homogenous patterns of social responsibility perception using the scores from the eight factors of VSRoSE. After the groups were identified using cluster analysis, we investigated whether these groups of students with different baseline scores might have reacted differently to the intervention. That is, we first identified different groups of students sharing similar entry characteristics (using the sub-scores of VSRoSE) and whether the intervention effects varied across these different groups of students. The guiding research questions are as follows.

RQ1. What are the effects of the ENACT program on engineering students’ social responsibility and willingness to act?

1-1. Is there a change in engineering students’ social responsibility after participating in the ENACT intervention program?
1-2. Is post-program social responsibility associated with students' willingness to act?

RQ2. What are the relationships between engaging in the ENACT program and different profiles of engineering students clustered based on their baseline social responsibility scores?

2-1. How many profiles emerge when engineering students are clustered based on their baseline social responsibility score?
2-2. Are there any differences in the effects of ENACT program among these groups?

## Theoretical frameworks

### The social responsibility of STEM professionals

As societal and global issues caused by advances in science and engineering increase, so does the need to enhance the social responsibility of STEM professionals. The definition of social responsibility has been an ongoing discussion, but it has been commonly accepted that social responsibility is distinguished from individual research ethics (e.g., honesty, integrity, rigorous process, collegiality, and respect) and embraces broader ethical considerations and behaviors for achieving the larger common good of humans, society, and the environment (Bielefeldt, 2018; Pimple, 2002; Wyndham et al., 2015). It also resonates with policy agendas, such as responsible research and innovation (RRI), which emphasizes that the goals of advanced research and innovation must align with important societal values and the sustainability of the environment (von Schomberg, 2013).

Despite the ambiguity and diversity of its definition, some scholars have proposed several dimensions of social responsibility in science and engineering as critical elements to consider. The dimensions of social responsibility that have most commonly been shared among scholars have been considerations of human welfare and safety, environmental sustainability, and societal consequences during science and engineering practices. This means that STEM professionals should anticipate possible risks in advance and actively take action to mitigate these risks for human beings, the environment, and society (Bielefeldt, 2018; Biswas, 2012; Glerup & Horst, 2014; Vanasupa et al., 2006; Wyndham et al., 2015).

Another common dimension of social responsibility includes the proactive consideration of societal needs and demands during the practices and pursuit of the common good. In other words, STEM professionals need to recognize, communicate, and reflect on a wide range of stakeholders, including marginalized groups, regarding their needs and expectations in order to balance potential interests or values (Lambrinidou & Canney, 2017; Stilgoe et al., 2014; Tassone et al., 2018). They should also conduct scientific and technological research not only in the direction of pursuing the interests of individuals or of the organization to which they belong, but also in the direction in which the benefits can be distributed to more people (Pimple, 2002).

Although this has been less widely accepted than other views (Schlossberger, 2016), some scholars have emphasized a more active social engagement as a major dimension of social responsibility, such as in public service, civic engagement, or deliberation of policies in related fields. For instance, Canney and Bielefeldt (2015) underlined the importance of active communication with the public on new scientific findings and sharing expertise to collaboratively solve societal and global issues. Similarly, the physicist Sakharov (1981), a Nobel Peace Laureate, emphasized the responsibility of informing and communicating with the public on the positive and negative consequences of scientific development by distributing scientific knowledge or warning of potential dangers. Schlossberger (2016), Bielefeldt (2018), and Glerup and Horst (2014) included pro bono activities such as volunteering to provide free engineering services for the community and voting or raising awareness as representatives on engineering-related issues and advisory boards for policymaking.

Integrating these perspectives in the field, Ko et al. (2021) recently developed VSRoSE, a measure of social responsibility that can be used for scientists and engineers in practice and training (i.e., college students in STEM majors). It consists of eight factors: concern for human welfare and safety (HUMAN), concern for environmental sustainability (ENVIR), consideration of societal risks and consequences (CONSEQ), consideration of societal needs and demands (NEEDS), pursuit of the common good (COMGOOD), civic engagement and services (CIVIC), communication with the public (COMMU), and participation in policy decision-making (POLICY). Since VSRoSE widely covers the scope of the social responsibility, we used it to test the effectiveness of our intervention.

### Educational efforts to enhance social responsibility

There has been little awareness of social responsibility among STEM professionals, and it has mostly been limited to issues involving individual research ethics. Glerup et al. (2017), for example, interviewed 29 scientists in emerging science technologies from different countries and found that the scientists considered social responsibility the lowest priority in their practices. Most of the professionals they interviewed mentioned “responsibility”, but their understanding of the concept and related practices was limited to the process of ensuring robust scientific research. Some scientists complained that RRI or similar policy agendas were imposed by parties with an insufficient understanding of scientific practices, and thus often irrelevant to their daily practice. Mejlgaard et al. (2019) also conducted interviews and a review of academic and policy documents to examine why scientists and engineers do not pay attention to teaching social responsibility for STEM practitioners. They found an implicit culture that regarded RRI as a subsidiary element of STEM education or as a level of common sense that STEM practitioners naturally acquire over time. They also reported the resistance of instructors to teach interdisciplinary aspects (e.g., science and technology sociology, and ethics).

Few educational programs for social responsibility have been implemented, and the evidence for their efficacy has not been strong (Zandvoort et al., 2013). There have only been a few well-designed programs, and these were developed for college students or adults in the STEM fields (Mejlgaard et al., 2019; Payne & Jesiek, 2018; Tassone et al., 2018; Zandvoort et al., 2013). The low level of social responsibility reported by the studies described above calls for the development of more in-service and pre-service training programs to enhance social responsibility.

Scholars have suggested some essential ideas of how to reconstruct curricula for college students in STEM to enhance their social responsibility, which can be summarized into two assertions: the program must have content that enhances the awareness of the interdisciplinary nature of science and technology. Zandvoort et al. (2013) collected good examples of empirical studies that enhanced social responsibility in different European countries and summarized them into shared visions on how to adequately equip STEM students with heightened social responsibility. They suggested the importance of understanding the interdisciplinary nature of the problems caused by science and technology, and the effective use of case studies, relevant projects, and community work to solve problems and mitigate risks (see pp. 1416–1417 for more details). They indicated that students should obtain a broad range of knowledge, not just limited to disciplinary knowledge, that includes sociology, history, and political science, so they can understand and communicate how science and technology have developed in society, how their development can cause risky problems, and how to deal with these problems. Seeing inequality, social injustice, and capitalism embedded in the advances in science and technology often motivated students to feel a responsibility towards SSIs and to act on resolving these issues (Bencze, 2017). Payne and Jesiek (2018) also suggested transdisciplinary models for increasing competencies of engineering students. They criticized engineering education, which mostly focused on technical problem solving by marginalizing the importance of knowledge production outside of academia, which could provide potentially relevant perspectives for engineering problem-solving. They applied the transdisciplinary model to engineering courses and observed that students showed increased positive outcomes. Their transdisciplinary model is quite convincing and is in line with the main intent of the ENACT model in the study.

The other key assertion is that the program must provide the opportunity to directly engage with the community and collaborate with diverse stakeholders through service learning or volunteering in community projects. Downey et al. (2006) insisted that collaborating with diverse groups of people is especially important. They felt that such collaboration helps engineers understand that engineering problems can be defined in different ways and can help them to see or accept conflicting perspectives as natural and legitimate differences. Other scholars have also agreed with this. Tassone et al. (2018) emphasized the creation of critical and constructive dialogical spaces where students are involved in knowledge co-creation/construction through reciprocal interaction with societal actors. Similarly, Bielefeldt and Canney (2016) highlighted the importance of students’ direct experience of community involvement. They conducted a survey to examine how engineering students viewed their responsibility and whether their views changed over time. They found that the students who showed an increase in social responsibility had a variety of college experiences such as community service and volunteer activities. In addition, it turned out that the students became more aware of the benefits of engineering for the community, and such awareness motivated them to do community service.

While the studies above provide important information on the key elements for effective education programs, they are somewhat limited to guiding the instructors to plan and lead a semester-long class and align their content with the principles of social responsibility. Even if instructors are willing to apply the above elements in their classes, they will still need a more concrete roadmap and guidance. A structured program can help the instructors to incorporate and sequence key elements to address social responsibility while meeting the pedagogical objectives of their individual courses for the semester. The ENACT program, which will be discussed in the next section, aims to meet such critical needs of instructors by providing a comprehensive instructional framework.

### The ENACT model as a new approach

The ENACT model (Lee et al., 2020) was developed through extensive literature reviews on education in science, technology, and engineering, RRI, and science and technology studies, and included expert consultation, along with an analysis of relevant educational programs. The ENACT model shares many commonalities with previous curriculum reform efforts for promoting engineers’ social responsibilities. However, it also has some unique features. It places a strong emphasis on students’ epistemological understanding of science, technology, and engineering. By linking socioscientific issues (SSIs) education with engineering education, students are encouraged to think deeply about why scientific and technological advances cannot avoid producing social and ethical issues. Based on the resulting SSI awareness, they were then asked to come up with solutions through scientific and engineering practices. In addition, the ENACT model provides specific instructional scaffolds to follow (e.g., stakeholder maps, futures wheel, and research methodologies), thus instructors can easily apply the model to their courses without extensive training or creating their own instructional devices.

Engineering courses commonly use societal problems or community issues, but SSIs used in the ENACT project have some distinctive features. Because SSIs refer to the social, ethical, and moral issues caused by the rapid development of science and technology (Zeidler et al., 2005), they represent well the complexity, uncontrollability, and uncertainty embedded in science, technology, and engineering. In the field of science education, SSIs have been addressed as an interdisciplinary approach for educating students to be responsible and scientifically literate citizens. Many empirical studies have proven the efficacy of SSIs in enhancing students’ understanding of the nature of science, technology, and engineering (Lee & Lee, 2021) and in promoting their feelings of responsibility and willingness to act on the issues as proactive agents (Choi & Lee, 2021; Kim & Lee, 2021; Lee & Lee, 2021; Lee et al., 2013). Some exemplary programs implemented by the European Union or Canada, such as PARRISE (Promoting Attainment of Responsible Research and Innovation in Science Education) (Amos & Levinson, 2019; Ariza et al., 2021; Levinson, 2018), RISKEDU (Schenk et al., 2019; Wojcik et al., 2019), and STEPWISE (Science & Technology Education Promoting Wellbeing for Individuals, Societies & Environments) (Bencze, 2017), aim to promote students’ social responsibility as citizens and their activism in the context of SSIs.

Thus, the ENACT model begins with students identifying the SSIs that they want to explore. As shown in Fig. 1, the ENACT model includes two cycles. Cycle I (Steps 1, 2, & 3) aims to alter epistemological beliefs on science and technology development by identifying SSIs and examining various aspects of the issues (i.e., social implications, multiple stakeholders, moral & ethical aspects, complexity & uncertainty). Then, in Cycle II (Steps 4 & 5), the students engage in the problem-solving process by adopting scientific and engineering practices. In the process, they are encouraged to consider RRI values (i.e., responsive, inclusive, reflexive, and sustainable) to pursue more inclusive and sustainable development (Owen et al., 2013; Stilgoe et al., 2013; Tassone et al., 2018) and finally lead to responsible actions (e.g., community service and outreach activities). Through these steps, the students become more aware of the importance of cultivating social responsibility.

The ENACT program follows five stages that guide instructors and students and provides various strategies to complete each stage. In Stage 1, “Engage in SSIs”, STEM students explore and identify SSIs in their field of study or in topics of interest to them. Several scholars (Burdinger & Burdinger, 2006; Davis, 1999) have emphasized identifying and defining issues prior to problem solving because how an issue is defined (framing) can determine the level of motivation and behavior for problem solving (Bencze & Krstovic, 2017; Mueller & Zeidler, 2010). Stage 2, “Navigate SSIs”, is a stage in which an issue of interest is researched in earnest from various angles. In Stage 3, “Anticipate consequences”, students work on finding a direction to pursue sustainable development by predicting the social impact and potential risk factors of science and technology that may occur in the future. Stage 4, “Conduct scientific and engineering practices”, is a stage in which students implement a possible plan to resolve the issue. Students can use scientific inquiry or engineering methods (e.g., experimentation, simulation, and argumentation; Zafrani & Yarden, 2017) and programming by using programs such as Python and Matlab. Students can also create exhibits on high-tech research, such as nanoscience and renewable energy (Gorghiu et al., 2015). The focus is on reflecting so that the process of execution becomes a responsive, inclusive, reflexive, and sustainable process (see the content of Cycle II). Last, Stage 5, “Taking societal actions”, is a stage in which the derived solution is shared with colleagues and local communities, and various methods of participation and practice are carried out for sustainable social development. The ENACT model can be easily adapted to any class, as long as instructors are flexible in the choice of content foci and pedagogical approach (e.g., creative engineering design or research methodology courses). It also can be plugged into content-specific, project-based courses in which students can address societal problems in their projects.

## Methods

### Participants

Forty-six college students majoring in safety engineering at a university located in a southern metropolitan area of South Korea participated in this study. Initially, 49 participants were enrolled in the course Creative Engineering Design, which is one of the foundational courses for first-year students, but we excluded three students who rarely participated in the course. This course aims for an understanding of the purpose and principles of engineering, where various experiments are conducted to enhance design thinking and engineering tools are utilized. Most of the participating students were first-year students (*N* = 39, 84.8%) and were male (*N* = 33, 71.7%). They were assigned to nine groups, and each group consisted of five to six students. Since we did not have a comparison group, we selected a single group pre- and post-test design for our study, where participants were carefully measured before and after the implementation with the aim of verifying the representativeness of the participants of this study. Thus, we compared the questionnaire scores with the group of 606 STEM college students who had responded to the same questionnaire (VSRoSE) as part of developing and validating it (Ko et al., ). After comparing the mean scores in the pre-test between participants in this study and the original large group, we found that there were no statistically significant differences between the groups in almost all factors of the VSRoSE except for two factors: COMGOOD and COMMU.

### Intervention: the ENACT program

#### Implementation

The instructor, one of the co-authors, implemented the ENACT model-based course for the participants for 15 weeks. At each stage of ENACT, instructional strategies were used to scaffold the students’ learning and thus guided them to complete the goals of each stage. In Stage 1, the students selected either a specific technology (e.g., electric transportation or new materials such as plastic) that causes or has caused societal problems or an SSI that they researched using the internet or other media (e.g., personal information leakage, copyright infringement, sustainability of nuclear power plants, anonymity in internet spaces, or mask waste) of their interest. While researching the technology or the issue, the students in the group identified the main debatable aspects and discussed why they need to pay attention to this issue. In Stage 2, we introduced stakeholder maps as an instructional strategy for presenting the complexity and uncertainty of these issues (Bencze & Krostovic, 2017; Newton & Zeidler, 2020; Zeidler et al., 2019). For this, students identified major stakeholders related to the technology or issue they had chosen using the map and made connections among the stakeholders. Completing the map helped them understand that science or technology is not an independent entity but a complex system that connects various actors including humans and other organisms and systems (e.g., the environment, animals and plants, and other technologies; Latour, 2005) and helped them see the complexity and uncertainty inherently embedded in them. In Stage 3, we adapted the futures wheel (Glenn, 1994; Oviawe et al., 2021) and scenario methods (Levrini et al., 2019; Tasquier et al., 2019). Future anticipation has been suggested as an important process of RRI (Tassone et al., 2018). It does not indicate an accurate prediction of the impact of science and technology on society, but rather an exploration of the various ways that science and technology may have an impact on humans, the environment, or society. Using the futures wheel, the students visualized the primary, secondary, and tertiary effects that science and technology can have, and based on this, they designed scenarios depicting the probable future and the desirable future. Students also came up with ideas on how to reduce the gap between the two futures. This became a starting point for problem solving. Throughout Stages 1–3, they became aware of the nature of science and technology, namely, social implications, multiple stakeholders, moral and ethical aspects, and complexity and uncertainty (see the contents of Cycle I).

In Stage 4, the students started by clarifying the issues into a problem that they could solve. Once the focus of the problem was clearly defined, they came up with possible ideas to solve the problem by using brain writing and evaluating the ideas to choose the best solution using the advantage, limitation, and unique qualities (ALU) method; pair ranking method (PRM); or weighting-factored evaluation method (WFEM). They then designed and conducted their own studies using prototyping, scientific experiments, data analysis/visualization, surveys, or literature reviews. Some of the groups interviewed main stakeholders to better identify their needs. Last, in Stage 5, the students planned how to share their research findings. Because students were able to experience the importance of civic engagement as future engineers, they had opportunities to reflect on their social responsibility and roles. Some groups created brochures, posters, or education materials (e.g., cartoons) to share useful information and held campaigns to directly engage with the general public. One group visited senior community centers and donated newly designed masks. Over Stages 4 and 5, students were encouraged to consider major elements of RRI ideas (see the inside of Cycle II). An example of the ENACT program using the topic of electric scooters is summarized in Table 1.

**Table 1:** An example of the ENACT program

Stages	Instructional strategies	Examples of activities
1. Engage in SSIs	Internet search, discussion	Finding SSIs that result from cutting-edge science and technology Searching for news articles, videos, and other media resources on SSIs Selecting one issue (e.g., unable to hear e-scooters as they typically make little to no noise while moving) and investigating the science and technology that has brought about this controversial issue
2. Navigate SSIs	Stakeholder map	Identifying relevant and diverse stakeholders (e.g., e-scooter users, pedestrians, the visually impaired, road traffic authorities, and ride-sharing companies) Exploring the various perspectives of each stakeholder Visualizing the relationships among these stakeholders into a map
3. Anticipate consequences	Futures wheel, future scenario	Brainstorming to uncover the multiple levels of future consequences resulting from the issue Developing plausible and desirable future scenarios (anticipate a future where the problem continues; establish a vision when the problem is solved)
4. Conduct scientific and engineering practices	ALU, PRM, WFEM Experiments, prototyping, data analysis	Specifying the problem and coming up with multiple ideas to solve this problem (e.g., signals such as warning lights/sounds, a sensor buzzer that identifies that an e-scooter is approaching) Evaluating possible solutions (ALU, PRM, WFEM) Conducting scientific/engineering practices (e.g., prototypes, experiments, engineering design, or big data analysis)
5. Take action	Campaign, policy proposal	Taking action to solve the problem Implementing campaigns (e.g., social media news, video production, pamphlets, or citizen science)

#### Learning environment

Due to COVID-19, we provided classes mostly through Zoom to explain each stage of the ENACT model and guided students on how to proceed on their own project. Besides the Zoom classes, we mentored the students in groups to support them in completing their projects. The students used a workspace on the ENACT project website. The workspace was designed for students to complete the project following the steps. Each step consisted of several activities which scaffolded them to effectively fulfill each step. We adapted the self-determination theory (Deci & Ryan, 2000) and the achievement goal orientation theory (Linnenbrink-Garcia & Patall, 2015; Schunk et al., 2014) when designing the workspace. Self-determination theory emphasizes support for students' sense of autonomy (i.e., voluntary decision and participation), competence (i.e., growth), and relatedness (i.e., belongingness). Achievement goal orientation theory emphasizes the importance of mastery goal orientation (i.e., self-improvement, growth, learning, and discovery), rather than performance goal orientation (i.e., social competition and work for the evaluation rather than learning/growth). Because both theories offer strong support for the use of meaningful tasks, the use of socioscientific issues serves as a strong motivational catalyst. In addition, the ENACT instructional model explicitly emphasizes respect and empowerment of the students by letting them choose a topic of their own interest (support for autonomy and emphasis of mastery goal orientation) and develop it into a collaborative project with their peers (support for relatedness and emphasis of mastery goal orientation, discouraging performance goal orientation), thus making the project more personally meaningful to them (Marsh, 1990a, 1990b, 1993; Marsh et al., 2005). Faculty provided consistent scaffolding and frequent feedback to the students throughout the semester (support for competence and mastery goal orientation). Due to space limitations, we will not elaborate further, but these pedagogical strategies are supported by multiple contemporary motivation theories such as expectancy-value theory (Eccles & Wigfield, 2002) and self-efficacy theory (Bandura, 2001).

### Data collection

#### Students’ responses to the views of social responsibility of scientists and engineers

To measure students’ changing views of the social responsibility of scientists and engineers before and after the intervention, we used a scale based on VSRoSE (Ko et al., 2021). VSRoSE reflects a broadened perspective on the social responsibility of scientists and engineers and was developed through a rigorous validation process for college students in STEM fields (see the detailed validation process in Ko et al. (2021)). The version used in the current study was identical to the published version except for two items that were worded slightly differently.

The scale consists of thirty 5-point Likert-type scale items with eight factors: (1) concern for human welfare and safety; (2) concern for environmental sustainability; (3) consideration of societal risks and consequences; (4) consideration of societal needs and demands; (5) pursuit of the common good; (6) civic engagement and services; (7) communication with the public, and (8) participation in policy decision-making. The responses from VSRoSE were converted from Likert-type scale responses ranging from *strongly disagree* to *strongly agree* to numerical values from 1 to 5 (1 = *strongly disagree*, 5 = *strongly agree*). The internal consistency reliability was a Cronbach’s ɑ = .912, which is acceptable as shown in Table 2.

**Table 2:** Internal consistency reliability of the VSRoSE

Factors	Items	Reliability
1. Concern for human welfare and safety (HUMAN)	1–5	.771
2. Concern for environmental sustainability (ENVIR)	6–8	.773
3. Consideration of societal risks and consequences (CONSEQ)	9–13	.819
4. Consideration of societal needs and demands (NEEDS)	14–16	.756
5. Pursuit of the common good (COMGOOD)	17–19	.781
6. Civic engagement and services (CIVIC)	20–24	.767
7. Communication with the public (COMMU)	25–27	.837
8. Participation in policy decision-making (POLICY)	28–30	.746
Total (30)	1–30	.912

### Questionnaire for measuring the willingness to act

Based on the sociocognitive theory (Bandura, 2001) and empirical studies (e.g., Crall et al., 2013), we assumed that the students’ views on the social responsibility of STEM professionals could be closely linked to actual behaviors. Students with a high awareness of their role as STEM professionals are likely to present a higher willingness to take on the role. To support this assumption, we aimed to measure the degree to which students reported their willingness to act, and we conducted a correlation analysis between their willingness to act and the eight factors of VSRoSE. However, there was no existing measure that fit our purpose. Thus, we developed a survey that consists of three questions that ask students to mark the number of hours (0–10 h per week) that they are willing to participate in various programs to quantify their willingness. These programs would enhance (A) their research skills and content knowledge in the areas of their majors; (B) professional skills including communication, leadership, creativity, and business administration; and (C) participation in a project to solve SSIs (e.g., environmental pollution or toxic chemical reduction).

### Data analysis

To answer Research Question 1, we first conducted a repeated-measures analysis of variance (ANOVA) to investigate the efficacy of the ENACT program on college students. Since we used a pre-test/post-test design as quasi-experimental research, we compared the students’ mean pre- and post-test scores on each variable of VSRoSE (RQ 1–1). We also conducted a correlation analysis between students’ VSRoSE post-test scores and their willingness to participate in workshops, projects, and resolving SSIs (RQ 1–2).

For the Research Question 2, we first performed a Ward’s hierarchical cluster analysis using VSRoSE pre-test scores to identify student groups who had similar perceptions on the social responsibility of scientists and engineers (RQ 2–1). Cluster analysis was used to classify different objects into groups that are more similar to each other than the objects in other groups; specifically, Ward’s hierarchical cluster analysis was used to minimize within-group variation and maximize between-groups variation (Nulty, 2008). Increases to the within-cluster sum of squares index, dendrogram, and interpretability were used to determine the optimum number of clusters. To ease the understanding of the profile of the selected clusters, a graph for each cluster was presented along with descriptive statistics such as the mean and standard deviation of the VSRoSE sub-scales. The results from cluster analysis provided a way to test whether the efficacy of the ENACT program varied among clusters of students. Using the identified clusters as independent variables (between-group factor), we conducted multivariate ANOVAs (MANOVAs) with VSRoSE sub-scales as dependent variables (RQ 2–2). Post hoc tests were performed to determine which clusters showed the significant differences from each other. Wilcoxon signed-rank tests were also conducted to investigate the score changes before and after the ENACT program in each cluster.

## Results

Results are presented in the following two sections following the order of the research questions. This section offers the results of the analyses, aiming to answer research questions RQ 1–1 (Is there a change in engineering students’ social responsibility after participating in the ENACT intervention program?), RQ 1–2 (Is post-program social responsibility associated with students' willingness to act?), RQ 2–1 (How many profiles emerge when engineering students are clustered based on their baseline social responsibility score?), and RQ 2–2 (Are there any differences in the effects of ENACT program among these groups?).

### The overall effects of the ENACT program (RQ 1–1)

A repeated-measures ANOVA was conducted to investigate the efficacy of the ENACT program on students’ views of the social responsibility of scientists and engineers. As shown in Table 3, there were statistically significant increases in three factors. After the intervention, the participants demonstrated significantly higher perceptions on the consideration of societal needs and demands (NEEDS, *p* = .030), civic engagement and services (CIVIC, *p* = .010), and participation in policy decision-making (POLICY,* p* = .047). The mean scores of the other four factors (HUMAN, CONSEQ, COMGOOD, and COMMU) did not present significant increases. Although we only found positive changes in three factors of VSRoSE, the changes are very significant, considering that in previous studies, students’ social responsibility rarely developed over the school years without explicit education (e.g., Bielefeldt & Canney, 2016).

**Table 3:** Repeated measures ANOVA for VSRoSE

Factors	Pre		Post		*F* (1, 45)	eta-squared
	*M*	SD	*M*	SD		
HUMAN	4.30	0.467	4.29	0.519	.043	.001
ENVIR	4.36	0.574	4.21	0.499	4.340*	.088
CONSEQ	4.24	0.414	4.29	0.488	.887	.019
NEEDS	3.80	0.846	4.08	0.587	5.036*	.101
COMGOOD	3.91	0.610	4.08	0.593	2.674	.056
CIVIC	3.71	0.674	3.93	0.537	7.164*	.137
COMMU	4.23	0.592	4.23	0.553	.000	.000
POLICY	3.85	0.578	4.07	0.623	4.184*	.085
Total	4.05	0.426	4.15	0.457	2.429	.051

*p < .05

HUMAN: concern for human welfare and safety; ENVIR: concern for environmental sustainability; CONSEQ: consideration of societal risks and consequences; NEEDS: consideration of societal needs and demands; COMGOOD: pursuit of the common good; CIVIC: civic engagement and services; COMMU: communication with the public; POLICY: participation in policy decision-making

Notably, the mean scores of concerns for environmental sustainability (ENVIR) dropped significantly after the intervention (*p* < .043). The result was unexpected, but one possible interpretation of the drop in score could be the ceiling effect. The students already perceived the importance of protecting environmental sustainability before the intervention. They reported a mean score of 4.36 out of 5, which was the highest score among the eight factors.

### The association with students' willingness to act (RQ 1–2)

To explore the possibility that the improvement of participants’ social responsibility is derived from their behaviors towards solving SSIs, we examined the correlation between the post-test score of VSRoSE and willingness to act. Cognitive factors such as knowledge, perception, and preferences are often closely linked to actual behaviors (Crall et al., 2013; Yeh et al., 2021). Thus, we assumed that STEM college students with higher social responsibility were likely to have a higher willingness to act to resolve SSIs (willingness to act). Their willingness to act score was calculated as the number of hours per week they were willing to invest in participating in three programs: (A) to enhance their research skills and content knowledge in their major areas; (B) to enhance their professional skills including communication, leadership, creativity, and business administration; and (C) to volunteer to solve SSIs. As shown in Table 4, students’ social responsibility presented a higher correlation with their willingness to voluntarily participate in projects that can solve SSIs rather than their willingness to participate in cultivating competency or professional skills in their major (A, B). This suggests that cultivating social responsibility through the ENACT program can ultimately influence students to take more interest in and act on SSIs.

**Table 4:** Correlation between VSRoSE post-scores and willingness to act

Factors	A Capabilities in major	B Professional skills	C Solving SSIs
HUMAN	.139	.116	.207
ENVIR	.237	.285	.240
CONSEQ	.182	.241	.348*
NEEDS	.170	.212	.401**
COMGOOD	.247	.302*	.363*
CIVIC	.283	.239	.490**
COMMU	.227	.346*	.285
POLICY	.207	.257	.489**
TOTAL	.253	.286	.425**

*p < .05, **p < .01

(A) indicates a willingness to participate in a program that will enhance research skills and content knowledge in their major areas; (B) indicates a willingness to participate in a program that will enhance professional skills including communication, leadership, creativity, and business administration; and (C) indicates a willingness to volunteer for projects that will solve socioscientific issues

HUMAN: Concern for human welfare and safety; ENVIR: Concern for environmental sustainability; CONSEQ: Consideration of societal risks and consequences; NEEDS: Consideration of societal needs and demands; COMGOOD: Pursuit of the common good; CIVIC: Civic engagement and services; COMMU: Communication with the public; POLICY: Participation in policy decision-making

### The profiles of engineering students on the initial baseline VSRoSE scores (RQ 2–1)

**Fig. 2:**
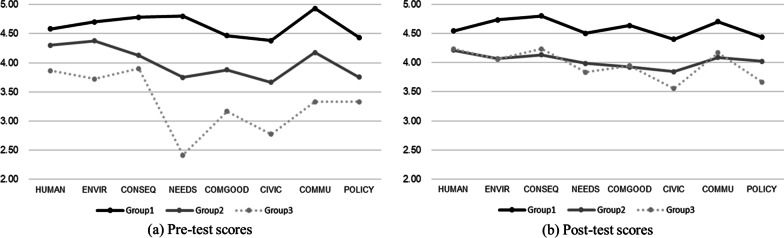
VSRoSE scores using a three-cluster model

A cluster analysis was used to identify the groups that showed different patterns in their VSRoSE scores and to examine the differences in the intervention effects among the groups. After thoroughly examining possible options between using two to four clusters, we selected the three-cluster solution from the dendrogram and interpretability to provide the best representation of the data. The three clusters were labeled with the names: Group 1 (High), Group 2 (Medium), and Group 3 (Low). Figure 2 shows the pre- and post-test scores by cluster.

We identified several interesting patterns by comparing Fig. 2a and b. First, Group 1, which had high VSRoSE scores in the pre-test, still showed high scores in every single factor in VSRoSE after the intervention. A Schéffé test shows that the post-test scores in the eight dimensions are significantly higher than the ones of Group 2 or Group 3 (see Table 5). Second, the distribution of Group 2’s scores for each dimension became smooth. In the pre-test, Group 2 showed relatively lower scores in NEEDs, CIVIC, and POLICY than other dimensions, but the scores of those three dimensions increased. Lastly, the most noticeable pattern was found in Group 3. Group 3 showed a low VSRoSE score in the pre-test, but the post-test scores increased and so presented a score pattern similar to that of Group 2. Post hoc tests for post-test scores in Table 5 show that the difference between Group 2 and Group 3 almost disappeared. This indicates that the ENACT program contributed more to increasing the social responsibility of the students in the lower group.

**Table 5:** MANOVA by group and post hoc results

Factors	Pre-test VSRoSE		Post-test VSRoSE	
	*F*	Schéffé	*F*	Schéffé
HUMAN	5.191*	1, 2 > 2, 3	1.559	1, 2, 3
ENVIR	6.894**	1, 2 > 3	9.77***	1 > 2, 3
CONSEQ	22.998***	1 > 2, 3	9.832***	1 > 2, 3
NEEDS	43.381***	1 > 2 > 3	3.961*	1 > 2, 3
COMGOOD	13.429***	1 > 2 > 3	7.080**	1, 3 > 2, 3
CIVIC	19.790***	1 > 2 > 3	7.239**	1 > 2, 3
COMMU	35.926***	1 > 2 > 3	5.573**	1, 3 > 2, 3
POLICY	11.596***	1 > 2, 3	3.364*	1, 2, 3

*p < .05, **p < .01, ***p < .001

HUMAN: Concern for human welfare and safety; ENVIR: Concern for environmental sustainability; CONSEQ: Consideration of societal risks and consequences; NEEDS: Consideration of societal needs and demands; COMGOOD: Pursuit of the common good; CIVIC: Civic engagement and services; COMMU: Communication with the public; POLICY: Participation in policy decision-making

### The differences in the effects of the ENACT program by group (RQ 2–2)

To examine the effects of the ENACT program within the group, we compared the pre- and post-test scores of each group. Group 1 maintained high scores across all the dimensions of VSRoSE after the intervention, but statistically significant changes were not observed. For Group 2, as shown in Table 6, the scores in NEEDS, CIVIC, and POLICY significantly increased after the intervention but the score for ENVIR decreased. For Group 3, the mean scores of all the dimensions increased to some extent, and the total VSRoSE score significantly increased in the post-test in Table 7. While the number of students in Group 3 was six (12.5%), which is quite small, we found that the scores in NEEDs and COMMU showed a statistically significant increase after the intervention.

**Table 6:** Repeated-measures analysis of variance for VSRoSE (Group 2, *N* = 30)

Factors	Pre		Post		*F*	eta-squared
	*M*	SD	*M*	SD		
HUMAN	4.30	0.457	4.21	0.543	.635	.021
ENVIR	4.38	0.538	4.07	0.450	17.277***	.373
CONSEQ	4.13	0.270	4.13	0.444	.009	.000
NEEDS	3.75	0.487	3.98	0.500	4.801*	.142
COMGOOD	3.88	0.442	3.92	0.558	.132	.005
CIVIC	3.67	0.364	3.84	0.438	4.762*	.141
COMMU	4.18	0.379	4.09	0.495	1.149	.038
POLICY	3.76	0.446	4.02	0.525	5.118*	.150
Total	4.01	0.210	4.04	0.398	.219	.008

*p < .05, ***p < .001

**Table 7:** Wilcoxon signed-rank test for VSRoSE (Group 3, *N* = 6)

Factors	Pre		Post		*Z*	eta-squared
	*M*	SD	*M*	SD		
HUMAN	3.87	0.393	4.23	0.266	1.890	.545
ENVIR	3.72	0.390	4.06	0.390	1.732	.500
CONSEQ	3.90	0.486	4.23	0.408	1.378	.347
NEEDS	2.42	0.736	3.83	0.516	2.214*	.765
COMGOOD	3.17	0.691	3.94	0.251	1.826	.605
CIVIC	2.78	0.841	3.56	0.360	1.841	.599
COMMU	3.33	0.558	4.17	0.658	2.060*	.708
POLICY	3.33	0.516	3.67	0.699	.680	.130
Total	3.37	0.249	3.96	0.279	2.207*	.834

*p < .05

HUMAN: Concern for human welfare and safety; ENVIR: Concern for environmental sustainability; CONSEQ: Consideration of societal risks and consequences; NEEDS: Consideration of societal needs and demands; COMGOOD: Pursuit of the common good; CIVIC: Civic engagement and services; COMMU: Communication with the public; POLICY: Participation in policy decision-making

## Discussion

This study explored to what extent the ENACT program contributed to enhancing college students’ views of the social responsibility of scientists and engineers. Previous studies (e.g., Bielefeldt & Canney, 2016) have reported the stability of STEM students’ social responsibility attitudes throughout their college years. Yet the implementation of the ENACT program showed students to have statistically significant changes in three of the factors (NEEDS, CIVIC, and POLICY) defined in VSRoSE. By engaging in the ENACT program, the college students became aware of the importance of recognizing a wide range of stakeholders, including marginalized groups and tried to communicate and reflect on their needs and expectations to balance potential interests or values (NEEDS). They also identified the need for volunteering to share their skills and knowledge, providing advice, performing pro bono activities, and collaborating with the public to solve community problems related to science and technology (CIVIC). They valued participation in policymaking (POLICY) to support the proposal or establishment of policies for the development of science and technology, the even distribution of its benefits, and attracting investment in related fields.

Several possible factors contributed to the positive changes. First, the participating students chose their topics for investigation based on their own interests. Some of the topics were connected to their academic majors and some were related to new technology or SSIs that they had encountered in their everyday lives. Personal meaningfulness on a given task often leads to intrinsic motivation for learning, and this motivation contributes to increasing autonomous engagement (Cordova & Lepper, 1996; Niemiec & Ryan, 2009). Thus, in Cycle I, the instructors allowed for a significant amount of time in finding topics and let students examine new technologies or SSIs from diverse angles. Second, several scaffolding approaches were used to support a sense of autonomy. For example, the ENACT online workspace included several guiding questions for each stage of ENACT that students responded to in completing the stages. On the workspace, they could upload what they did for each stage and check feedback from the instructor. Drawing stakeholder maps and futures wheels also scaffolded the students to examine the diverse stakeholders and their complex connections and to anticipate possible future scenarios. Similar studies such as Bencze and Krstovic (2017), Choi and Lee (2021), and Kim et al. (2021) reported that the process of identifying unethical, biased, or unreasonable elements among stakeholders could provide motivation to find solutions to SSIs autonomously. Last, we encouraged the students to meet various groups of people in their community because previous studies (Bielefeldt & Canney, 2016; Lathem et al., 2011; Tassone et al., 2018) had confirmed that service-learning or community engagement experiences contributed to the increase in a feeling of responsibility. In Cycle II, some students engaged in campaigns using brochures, visited senior centers to provide residents with newly designed masks, and surveyed the perceptions of the general public on these solutions.

The three groups of the participants with similar patterns of pre-test scores on VSRoSE, revealed by cluster analysis, were particularly encouraging. The results suggest that different approaches must be taken to support students in different clusters. The participants who showed higher social responsibility even before the intervention may not need further specific education for increasing social responsibility. However, participants with lower social responsibility need more systemic approaches like the ENACT program. The intervention showed the greatest effect in the group with the lowest VSRoSE score (Group 3). In the case of Group 2, which showed a medium level of scores, the three factors (NEEDS, CIVIC, and POLICY) that showed low scores before the intervention significantly improved after the intervention. Considering that their academic major of safety engineering is a field that explicitly involves diverse stakeholders and provides practical solutions to make citizens’ lives more convenient and safer, the increase in the three factors is particularly meaningful.

This study also showed that STEM students’ perceptions of social responsibility as scientists and engineers are interrelated with their willingness to resolve SSIs. This indicates that their experiences of engaging in the ENACT program contributed to increasing their interest in SSIs and motivation to participate in solving SSIs. Table 4 shows that students with high social responsibility tended to possess a greater willingness to solve SSIs than to improve their knowledge or professional skills related to their majors. In other words, in order to cultivate STEM professionals’ social responsibility, it is important to provide opportunities to understand the nature of science and technology and to take part in a project that they themselves initiate based on an epistemological understanding of science and technology.

Last, for this study, we implemented the ENACT model in a course on creative engineering design. This is a foundational course for first-year students in engineering fields. Most of the engineering schools in South Korea offer similar engineering design or research methodology courses. In these courses, SSIs are often treated as a problem requiring a quick fix through design changes or product development. However, this is a serious problem because these issues often involve uncertainty, uncontrollability, and complexity, which a quick fix cannot address. The ENACT program provides a critical opportunity for students to consider why the issues are raised in the first place, who the stakeholders are, and how a solution might have an impact on people, the environment, and society. By learning through each cycle of the ENACT program, the students can reflect on the nature of science and technology that caused SSIs and develop an informed solution. A natural extension of the current project is to implement the ENACT program in multiple courses in the STEM curriculum across the full duration of college education and track the long-term effects of the ENACT program after students enter the workforce.

## Conclusion and implications

In the paper, we have discussed the importance of enhancing the social responsibility of STEM professionals in practice and training. We also have shown that social responsibility can be nurtured by systemic instructional approaches, and increased social responsibility can lead to greater commitment to resolving SSIs. Since the current sample included only those students in safety engineering majors, we need to be careful not to overgeneralize these positive outcomes by applying them to other engineering fields. The effect of the ENACT program and the score patterns of social responsibility might vary depending on the field. However, when compared with the larger data from our earlier study (Ko et al., 2022), which involved 606 STEM college students from various science and engineering majors, the current sample (safety engineering students) turned out to be similar in six of the eight dimensions of VSRoSE. Thus, we are hopeful that the positive effects of the ENACT model will be extended to students in other fields of STEM, but future research should be conducted before a definitive conclusion can be drawn.

Mastering engineering content knowledge and skills is the key element of engineering curricula. However, as emphasized by many scholars, we need to use more holistic approaches to educate engineering students to be socially responsible (Lathem et al., 2011; Tassone et al., 2018; Vanasupa et al., 2006). In an era where capital drives the landscape of academic research in science and engineering fields [“academic capitalism”, as discussed by Hackett (2014, p. 635)], it is difficult for STEM professionals to prioritize social responsibility in their practice. However, for a sustainable society, we are compelled to incorporate social responsibility into the STEM curriculum. We believe that the ENACT model contributes toward this end.

As we emphasized throughout this paper, we face numerous societal and environmental issues and risks, directly and indirectly caused by science and engineering applications. In order to effectively cope with and control these risks, not only STEM professionals (practicing and in training), but also ordinary citizens should be part this endeavor. They must develop increased awareness of SSI-related issues, be able to make wise decisions over relevant government and corporate policies and practices, and be willing to collaborate with the experts to produce more sustainable solutions. For the reason, a fruitful next step will be to broaden the scope of the current research and apply the ENACT model to a wide range of college courses outside of STEM curricula. We believe that this type of citizenship course will be a great addition to the college core curriculum.

## Data Availability

The datasets generated and/or analyzed during the current study are not publicly available due to the IRB document statement but are available from the corresponding author on reasonable request.

## Abbreviations

SSIs: Socioscientific issues
STEM: Science, technology, engineering, and mathematics
ENACT: Engage in SSIs; Navigate SSIs; Anticipate consequences; Conduct scientific and engineering practices; Take action
VSRoSE: Views of Social Responsibility of Scientists and Engineers
